# Supplementary material for: COVID-19 vaccination coverage in deprived populations living in segregated colonies: A nationwide cross-sectional study in Hungary
Source: PLoS One. 2022 Feb 28;17(2):e0264363. doi: 10.1371/journal.pone.0264363 (PMC8884504; doi:10.1371/journal.pone.0264363)

PLOS ONE

COVID-19 vaccination coverage in deprived populations living in segregated colonies: a nationwide cross-sectional study in Hungary

--Manuscript Draft--

Manuscript Number:	PONE-D-21-32644
Article Type:	Research Article
Full Title:	COVID-19 vaccination coverage in deprived populations living in segregated colonies: a nationwide cross-sectional study in Hungary
Short Title:	COVID-19 vaccination coverage in segregated colonies of Hungary
Corresponding Author:	János Sándor, PhD<br>University of Debrecen Faculty of Medicine: Debreceni Egyetem Altalanos Orvostudomanyi Kar<br>Debrecen, HUNGARY
Keywords:	COVID-19 vaccination; Roma ethnicity; segregation; vaccination coverage; inequality
Abstract:	The largest ethnic minority in Hungary is Roma, comprising 8.7% of the population. A quarter of them live in segregated colonies (SCs). Their health care use is restricted in many respects. Whether fair COVID-19 vaccination coverage has been achieved in Hungary is accompanied with fair effectiveness in SCs, has not yet been studied. Using census data, the vaccination coverage in SCs and the complementary areas (CAs) in the same settlements of the country was determined. To describe the settlement level differences, the vaccination coverage (until June 30, 2021) in SCs were compared to those in CAs by age, sex, and eligibility for exemption certificate standardized measures. Aggregating settlement level data, the level of geographic discrimination in Hungary was also determined. According to nationwide aggregates, crude vaccination coverage was significantly lower in SCs (40.05%, 95% CI 39.87%-40.23%) than in CAs (65.42%, 95% CI 65.37%-65.46%). The relative standardized vaccination coverage was 0.643 (95% CI 0.639-0.647) in SCs. A total of 437 of the 938 investigated settlements showed significant local vaccination disparities. Hungarian SCs populated mainly by Roma are a distinct high-risk group with respect to COVID-19 vaccination. Special intervention adapted to SCs is needed to mitigate inequality in vaccination coverage and further consequences of the pandemic.
Order of Authors:	János Sándor, PhD
	Ferenc Vincze, PhD
	Maya Liza Shrikant
	László Kőrösi
	László Ulicska, PhD
	Karolina Kósa, PhD
	Róza Ádány, DSc
Additional Information:	
Question	Response
**Financial Disclosure**<br><br>Enter a financial disclosure statement that describes the sources of funding for the work included in this submission. Review the submission guidelines for detailed requirements. View published research articles from *PLOS ONE* for specific	This study was carried out in the framework of the “ Routine monitoring for the health status and health care use in the Hungarian segregated colonies” program (BM/6327-3/2021, FEIF/951/2021-ITM), supported by the Deputy State Secretariat for Social Inclusion, Ministry of Interior. (https://2010-2014.kormany.hu/en/ministry-of-interior)<br><br>JS, FV, LK, KK, RÁ received grant from that program.<br><br>The funder had no role in study design, data collection and analysis, decision to

Powered by Editorial Manager® and ProduXion Manager® from Aries Systems Corporation

examples.<br><br>This statement is required for submission and **will appear in the published article** if the submission is accepted. Please make sure it is accurate.<br><br>**Unfunded studies**<br>Enter: *The author(s) received no specific funding for this work.*<br><br>**Funded studies**<br>Enter a statement with the following details:<br>• Initials of the authors who received each award<br>• Grant numbers awarded to each author<br>• The full name of each funder<br>• URL of each funder website<br>• Did the sponsors or funders play any role in the study design, data collection and analysis, decision to publish, or preparation of the manuscript?<br>• **NO** - Include this sentence at the end of your statement: *The funders had no role in study design, data collection and analysis, decision to publish, or preparation of the manuscript.*<br>• **YES** - Specify the role(s) played.<br><br>* typeset	publish, or preparation of the manuscript.
**Competing Interests**<br><br>Use the instructions below to enter a competing interest statement for this submission. On behalf of all authors, disclose any competing interests that could be perceived to bias this work—acknowledging all financial support and any other relevant financial or non-financial competing interests.<br><br>This statement is required for submission and **will appear in the published article** if the submission is accepted. Please make sure it is accurate and that any funding sources listed in your Funding Information later in the submission form are also declared in your Financial Disclosure statement.	The authors have declared that no competing interests exist.

View published research articles from *PLOS ONE* for specific examples.<br><br>**NO authors have competing interests**<br><br>Enter: *The authors have declared that no competing interests exist.*<br><br>**Authors with competing interests**<br><br>Enter competing interest details beginning with this statement:<br><br>*I have read the journal's policy and the authors of this manuscript have the following competing interests: [insert competing interests here]*<br><br>* typeset	
**Ethics Statement**<br><br>Enter an ethics statement for this submission. This statement is required if the study involved:<br><br>• Human participants<br>• Human specimens or tissue<br>• Vertebrate animals or cephalopods<br>• Vertebrate embryos or tissues<br>• Field research<br><br>Write "N/A" if the submission does not require an ethics statement.<br><br>General guidance is provided below. Consult the submission guidelines for detailed instructions. **Make sure that all information entered here is included in the Methods section of the manuscript.**	Data provided by the Hungarian NHIF were used in this study. All data processed in our secondary analyses were geographically aggregated. Because individual information was not used, ethical approval and written informed consent were not required according to the Hungarian legal framework. The protocol to produce segregation-specific indicators was approved by the Office of the Commissioner for Fundamental Rights (AJB-3147/2013), the general director of the NHIF (E0101/215-3/2014), and the Hungarian National Authority for Data Protection and Freedom of Information (NAIH/2015/826/7N).

**Format for specific study types**<br><br>**Human Subject Research (involving human participants and/or tissue)**<br>• Give the name of the institutional review board or ethics committee that approved the study<br>• Include the approval number and/or a statement indicating approval of this research<br>• Indicate the form of consent obtained (written/oral) or the reason that consent was not obtained (e.g. the data were analyzed anonymously)<br><br>**Animal Research (involving vertebrate animals, embryos or tissues)**<br>• Provide the name of the Institutional Animal Care and Use Committee (IACUC) or other relevant ethics board that reviewed the study protocol, and indicate whether they approved this research or granted a formal waiver of ethical approval<br>• Include an approval number if one was obtained<br>• If the study involved *non-human primates*, add *additional details* about animal welfare and steps taken to ameliorate suffering<br>• If anesthesia, euthanasia, or any kind of animal sacrifice is part of the study, include briefly which substances and/or methods were applied<br><br>**Field Research**<br><br>Include the following details if this study involves the collection of plant, animal, or other materials from a natural setting:<br>• Field permit number<br>• Name of the institution or relevant body that granted permission	
**Data Availability**<br><br>Authors are required to make all data underlying the findings described fully available, without restriction, and from the time of publication. PLOS allows rare exceptions to address legal and ethical concerns. See the PLOS Data Policy and FAQ for detailed information.	No - some restrictions will apply

A Data Availability Statement describing where the data can be found is required at submission. Your answers to this question constitute the Data Availability Statement and **will be published in the article**, if accepted.<br><br>**Important:** Stating 'data available on request from the author' is not sufficient. If your data are only available upon request, select 'No' for the first question and explain your exceptional situation in the text box.<br><br>Do the authors confirm that all data underlying the findings described in their manuscript are fully available without restriction?	
**Describe where the data may be found in full sentences. If you are copying our sample text, replace any instances of XXX with the appropriate details.**<br><br>• If the data are **held or will be held in a public repository**, include URLs, accession numbers or DOIs. If this information will only be available after acceptance, indicate this by ticking the box below. For example: *All XXX files are available from the XXX database (accession number(s) XXX, XXX.).*<br>• If the data are all contained **within the manuscript and/or Supporting Information files**, enter the following: *All relevant data are within the manuscript and its Supporting Information files.*<br>• If neither of these applies but you are able to provide **details of access elsewhere**, with or without limitations, please do so. For example:<br><br>*Data cannot be shared publicly because of [XXX]. Data are available from the XXX Institutional Data Access / Ethics Committee (contact via XXX) for researchers who meet the criteria for access to confidential data.*<br><br>*The data underlying the results presented in the study are available from (include the name of the third party*	The data underlying the results presented in the study are available from János Sándor upon request (janos.sandor@med.unideb.hu).

Powered by Editorial Manager® and ProduXion Manager® from Aries Systems Corporation

and contact information or URL).<br>• This text is appropriate if the data are owned by a third party and authors do not have permission to share the data.<br><br>* typeset	
Additional data availability information:	Tick here if the URLs/accession numbers/DOIs will be available only after acceptance of the manuscript for publication so that we can ensure their inclusion before publication.

Manuscript Click here to access/download;Manuscript;Manuscript.docx

COVID-19 vaccination coverage in deprived populations living in segregated colonies: a nationwide cross-sectional study in Hungary

János Sándor PhD[1*], Ferenc Vincze PhD[1], Maya Liza Shrikant[1,2], László Kőrösi[3], László Ulicska PhD[4], Karolina Kósa PhD[5], Róza Ádány DSc[1,6]

[1] Department of Public Health and Epidemiology, Faculty of Medicine, University of Debrecen, Debrecen, Hungary

[2] Arizona State University, Tempe, Arizona, U.S.A.

[3] National Health Insurance Fund, Budapest, Hungary

[4] Deputy State Secretariat for Social Inclusion, Ministry of Interior, Budapest, Hungary

[5] Department of Behavioral Sciences, Faculty of Medicine, University of Debrecen, Debrecen, Hungary

[6] MTA-DE Public Health Research Group, Department of Public Health and Epidemiology, Faculty of Medicine, University of Debrecen, Debrecen, Hungary

* Corresponding author

E-mail: janos.sandor@med.unideb.hu (JS)

Abstract

The largest ethnic minority in Hungary is Roma, comprising 8.7% of the population. A quarter of them live in segregated colonies (SCs). Their health care use is restricted in many respects. Whether fair COVID-19 vaccination coverage has been achieved in Hungary is accompanied with fair effectiveness in SCs, has not yet been studied. Using census data, the vaccination coverage in SCs and the complementary areas (CAs) in the same settlements of the country was determined. To describe the settlement level differences, the vaccination coverage (until June 30, 2021) in SCs were compared to those in CAs by age, sex, and eligibility for exemption certificate standardized measures. Aggregating settlement level data, the level of geographic discrimination in Hungary was also determined. According to nationwide aggregates, crude vaccination coverage was significantly lower in SCs (40.05%, 95% CI 39.87%-40.23%) than in CAs (65.42%, 95% CI 65.37%-65.46%). The relative standardized vaccination coverage was 0.643 (95% CI 0.639-0.647) in SCs. A total of 437 of the 938 investigated settlements showed significant local vaccination disparities. Hungarian SCs populated mainly by Roma are a distinct high-risk group with respect to COVID-19 vaccination. Special intervention adapted to SCs is needed to mitigate inequality in vaccination coverage and further consequences of the pandemic.

Introduction

The SARS-CoV-2 pathogen brought on a serious pandemic, which has been forecasted for a long time. [1-3] The echo of the concerned is reflected in the dramatic health, economic and social loss caused by the first three waves of the COVID-19 pandemic affecting all countries of the world. [4] The profound inequalities with respect to infection rate [5], mortality rate [6-11], and vaccination coverage are [12] among the dominant characteristics of this pandemic.

When and where COVID-19 vaccines became available, and the vaccination programs' target groups were determined by taking into consideration the susceptibility of individuals to lethal complications (e.g., elderly individuals, patients with chronic diseases) and to the personnel involved in COVID-19 patients' health care. [13]

The distinct (i.e., socioeconomic status independent) risk factor role of race/ethnicity has been demonstrated by investigations based on person-level race/ethnicity classification. [14-16] Studies, both on migrants by their country of birth [17] and geographical analyses of aggregated data on racial/ethnic composition and vaccination coverage [18-23], confirmed this observation. Therefore, strict monitoring of the racial/ethnic dimension of vaccination inequalities is urgently needed. [24,25] To date, there are published reports on successful interventions aimed at diminishing racial/ethnic disparities in vaccination coverage. [26-28]

These studies suggest that vaccination programs should consider racial/ethnic minorities as special high-risk groups that require minority-adapted approaches (complementing the main programs designed for the majority) to diminish the consequences of the vaccine-preventable COVID-19 pandemic in the forthcoming wave(s) of the pandemic.

Central and Eastern European (CEE) countries are home to 10-12 million Roma (many of them in segregated colonies, SC) and they are the largest ethnic minority in Europe. Unfortunately, there are no socioeconomic status (SES) or ethnic minority-specific monitoring programs either for COVID-19 health impacts or COVID-19 vaccination

effectiveness. Although it seems probable that the CEE Roma are similar to the Black populations in the US and UK with respect to health care use, the lack of monitoring prevents even planning targeted pandemic intervention. Taking into consideration disadvantaged SES and the health status of Roma in CEE countries with limited access to health care [29], the COVID-19 vaccination coverage among this people group needs to be evaluated.

In Hungary, the last census in 2011 classified 315,583 persons as Roma (3.18% of the total population) by self-declaration. This approach (due to the negative historical experiences of ethnic minorities in governmental registration) seriously underestimates the number of Roma people. A special survey used the active contribution of the local municipalities estimated their number to be 870,000 (8.75% of the Hungarian population). [30,31]

The poorer health status of the Roma living in Hungary is well documented. [32,33] Approximately one-quarter of them [34] with a critical health status live in SCs. To improve the effectiveness of interventions targeting this high-risk population, a governmental decree (314/2012) defines the SCs utilizing census data. This establishes the production of SC-specific indicators by which the needs' exploration and the elaboration of interventions, as well as an assessment of the interventions' effectiveness can be carried out.

Roma comprised 94% of the inhabitants in SCs in 2005 [34]. Although the Roma characteristics of SCs have weakened due to social changes over the last two decades, there have been no new investigations on the proportion of Roma inhabitants in SCs.

The free-of-charge voluntary COVID-19 vaccination program has been organized by the government in Hungary and it started on 26 December 2020. By the time our dataset was generated on 30 June 2021, Hungarian vaccination coverage was at 58.89% among adults, the 2nd highest in Europe according to the European Centre for Disease Prevention and Control.

Our investigation utilized the special opportunity of the Hungarian health statistical system (e.g., its ability to produce SC-specific indicators), to describe COVID-19 vaccination

coverage in Hungary's SCs in relation to the nonsegregated part of the settlements to determine whether the population living in SCs dominated by Roma should be a distinct target group for vaccination programs.

Methods

Setting

This investigation encompassed the whole country. All COVID-19 vaccinations registered before 30 June 2021 were used in this study. The government organized a free-of-charge vaccination program using the Oxford/AstraZeneca COVID-19 vaccine, Janssen Ad26.COV2.S COVID-19 vaccine, Moderna COVID-19 (mRNA-1273) vaccine, Pfizer-BioNTech COVID-19 mRNA vaccine, Sinopharm COVID-19 vaccine, and the Sputnik V vaccine. The National Health Insurance Fund (NHIF) registered all vaccinations.

Mapping the segregated colonies

A governmental decree defines SCs as within settlement clustering of residents 18-59 years old with not higher than primary level education and a lack of work-related income. The Hungarian Central Statistical Office determines these clusters in SCs and the complementary areas (CA) of the settlements not belonging to any SC. Each Hungarian household is classified in this system as either an SC or a CA, in a mutually exclusive manner.

With the addresses of the adults the NHIF can define populations living in certain SCs or CAs. Therefore, all Hungarian adults can be classified as inhabitants living in an SC or living in a CA.

Data Sources

Data were provided by the NHIF to which reporting COVID-19 vaccinations is compulsory. The NHIF updated their database day-by-day. In this study, vaccinations until 30 June 2021 were included.

The NHIF characterized all patients by their age, sex and eligibility for an exemption certificate (deprived patients with chronic disease are supported by an exemption certificate which is released by the local municipality based on the recommendation of the patient's GP), and prepared the age group-, sex-, and eligibility for an exemption certificate-aggregated tables for each SC and CA based on the administration of the first and second COVID-19 vaccination doses.

The NHIF provided the target population data in the same structure. The age- (5-year bands), sex-, and possession of exemption certificate-specific numbers of vaccinations and populations were provided for each SC and CA.

Statistical Analysis

People who have received both doses of the vaccine are defined as 'vaccinated' in this analysis. The crude vaccination coverage (cVC) was calculated for each SC and CA. The different vaccine types were not distinguished. National age, sex, and eligibility for exemption certificate-specific reference vaccination ratios were calculated and used in indirect standardization. Age, sex, and exemption certificate standardized vaccination coverage (sVC) was computed for each SC and CA.

The settlement-specific sVC was evaluated by comparing to the local reference value of settlement-specific sVC in CAs. Relative standardized vaccination coverage (RsVC), the ratio of sVCs for the SC to sVCs for the CA of the same settlement, along with the corresponding 95% confidence interval (95% CI), were calculated. (In the settlements where more than one SC was located, settlement-specific aggregated SC measures were computed by summarizing the observed number of vaccinations, populations, and the expected number of vaccinations.) The excess number of vaccinations was calculated as the difference between the observed and the expected number of vaccinations. The proportion of vaccinations

attributable to segregation (attributable risk) was computed as the ratio of excess and observed number of vaccinations.

SPSS version 20 (IBM Corporation, New York, NY, USA) was used for the data analysis.

Ethical permission

Data provided by the Hungarian NHIF were used in this study. All data processed in our secondary analyses were geographically aggregated. Because individual information was not used, ethical approval and written informed consent were not required according to the Hungarian legal framework. The protocol to produce segregation-specific indicators was approved by the Office of the Commissioner for Fundamental Rights (AJB-3147/2013), the general director of the NHIF (E0101/215-3/2014), and the Hungarian National Authority for Data Protection and Freedom of Information (NAIH/2015/826/7N).

Results

There are 2006 SCs in Hungary belonging to 938 settlements. A total of 60.7% (4,987,661 persons) of the Hungarian adult population lives in settlements with SCs. Of the adults in the settlements with SCs, 5.6% live in a SC itself. This proportion was higher in the 18-59-year-old age group (6.6%) than in the older age group (3.45%), reflecting the poor life expectancy in SCs. Younger adults were overrepresented, and elderly adults were underrepresented in the SC population. (Fig 1) The proportion of adults who were eligible for an exemption certificate was 6.43% (N=17,814) in SCs and 2.39% (N=112,700) in CAs, showing a higher level of deprivation in the SC population ($p<0.001$ by the chi square test).

Fig 1. Demographic structure of the populations living in segregated colonies and complementary areas of the settlements with segregated colonies in Hungary.

The overall cVC was 64.0% (3,192,457 vaccinations). According to nationwide aggregates, cVC was significantly lower in SCs (40.05%, 95% CI 39.87%-40.23%) than in CAs (65.42%, 95% CI 65.37%-65.46%). The difference was much larger in the 18- to 59-year-old age group than in the older than 60 age group (Table 1).

Table 1 Crude COVID-19 vaccination ratios among adults in segregated colonies and in the complementary part of the settlements with segregated colonies (Hungary, as 30 June 2021)

Age groups	Number of observed cases in the segregated colonies	Target group in the segregated colonies	Vaccination coverage in the segregated colonies*	Number of observed cases in the complementary areas	Target group in the complementary areas	Vaccination coverage in the complementary areas*
**18-59 years**	75,567	219,156	34.48% [34.28%-34.68%]	1,888,229	3,094,472	61.02% [60.97%-61.07%]
**60+ years**	35,324	57,723	61.20% [60.80%-61.59%]	1,193,337	1,616,310	73.83% [73.76%-73.90%]
**18+ years**	110,891	276,879	40.05% [39.87%-40.23%]	3,081,566	4,710,782	65.42% [65.37%-65.46%]

*with 95% confidence interval

The sVC proved to be significantly lower in SCs (0.653, 95% CI 0.650-0.657) than in CAs (1.017, 95% CI 1.016-1.018). The RsVC was 0.643 (95% CI 0.639-0.647), associated with 58,807.9 missing vaccinations in SCs. Living in a SC accounted for 53% of the missing vaccinations. The difference was mainly generated in the 18-59-year-old age group, but it was significant in the older than 60-year-old age group as well (Table 2).

Table 2 Standardized COVID-19 vaccination coverage among adults in the segregated colonies and in the complementary part of the settlements with segregated colonies (Hungary, as 30 June 2021)

Age groups	Standardized vaccination coverage in segregated colonies*	Standardized vaccination coverage in complementary areas*	Relative standardized vaccination coverage in segregated colonies*	Number of excess cases in segregated colonies	Attributable risk in segregated colonies
**18-59 years**	0.593 [0.589-0.597]	1.026 [1.024-1.027]	0.578 [0.574-0.582]	-51,882.7	-68.7%
**60+ years**	0.836 [0.827-0.845]	1.003 [1.001-1.005]	0.833 [0.825-0.842]	-6,925.2	-19.6%
**18+ years**	0.653 [0.650-0.657]	1.017 [1.016-1.018]	0.643 [0.639-0.647]	-58,807.9	-53.0%

*with 95% confidence interval

The SC-specific RsVCs showed a wide distribution among adults (mean±SD: 0.771±0.251). The mean RsVC was lower for the 18-59-year-old age group (0.740±0.262) than for the older than 60-year-old adults (0.884±0.217). The majority of SCs had lower local reference sVCs compared to CAs (Fig 2).

Fig 2. Vaccination coverage among adults in segregated colonies compared to the complementary area of the settlements with segregated colonies in Hungary.

Mapping the SCs, where local interventions were established by sVC were significantly lower than the sVCs in the CAs in the corresponding settlement; the spatial distribution proved to be highly uneven (Fig 3a-b-c).

Fig 3a. Settlements with local intervention requirements in Hungarian counties established by low relative standardized vaccination coverage among 18- to 59-year-old

adults. (black square: standardized vaccination coverage in segregated colonies less than the complementary area's reference; white circle: standardized vaccination coverage in segregated colonies not deviating from the complementary area's reference)

Fig 3b. Settlements with local intervention requirements in Hungarian counties established by low relative standardized vaccination coverage among 60+ adults. (black square: standardized vaccination coverage in segregated colonies less than the complementary area's reference; white circle: standardized vaccination coverage in segregated colonies not deviating from the complementary area's reference)

Fig 3c. Settlements with local intervention requirements in Hungarian counties established by low relative standardized vaccination coverage among 18+ adults. (black square: standardized vaccination coverage in segregated colonies less than the complementary area's reference; white circle: standardized vaccination coverage in segregated colonies not deviating from the complementary area's reference)

Altogether, the number of SCs with significantly lower sVCs was 437, with 208,494 inhabitants, of which 80,065 were vaccinated. The number of missing vaccinations was 47,494.5. Most of the missing vaccinations were connected to the 18-59-year-old age group. In the 60+ age group, there were much fewer missing vaccinations (Table 3).

Table 3 Impact of segregation on COVID-19 vaccination coverage in high-risk segregated colonies (HRSC*)

Age groups	Number of HRSC	Population in HRSC	Number of vaccinations in HRSC	Number of excess cases in HRSC	Attributable risk in HRSC
**18-59**	412	159,433	52,578	-40,091.4	-76.3%

**years**					
**60+ years**	85	14,455	7,705	-2,862.6	-37.2%
**18+ years**	437	208,494	80,065	-47,494.5	-59.3%

*standardized vaccination coverage in the settlement's segregated colony significantly lower than that in complementary part of the same settlement

Discussion

Main findings

Our study shows that COVID-19 vaccination coverage is significantly lower in SCs than in CAs according to both cVC (40.05% vs. 65.42%) and sVC (0.653 vs. 1.017). This demonstrates that the Hungarian COVID-19 vaccination program did not avoid geographic inequality, which has a strong ethnic dimension. This observation confirms the reported experiences from other countries about the inequality-generating characteristics of COVID-19 vaccination programs.

The cVC was much higher and vaccination inequality was much less pronounced among elderly individuals older than 60 years, compared to adults in the 18-59 age range. Organizational efforts were much more intensive and started earlier among elderly adults than among younger adults. The aim of public health authorities is not only to reduce of the epidemic's intensity (reduction of susceptible population) but also to prevent serious complications, especially the infection of elderly individuals who were at higher risk. Personal motivation was also larger among elderly individuals for the same reasons.

By the time of our investigation, the sensitive population in the SCs was 165,988. If the vaccination coverage in SCs were equal to that in CAs, then there would be only 95,758 susceptible inhabitants in SCs. There are 1,795,204 adults without proper vaccination living in settlements with SC and 9.24% of them is from SCs. If the vaccination coverage in SCs were

equal to that in CAs, then 5.55% of the susceptible inhabitants would be from SCs. Consequently, the pandemic related future health loss can be prevented basically by the elevation of the vaccination in the CAs, but a not negligible risk reduction could be achieved by the enforced/adapted vaccination project in the SCs.

Our investigation described the huge variability of settlement-specific RsVC. SC-related under-vaccination was generated by 437 SCs, where the number of adult inhabitants was 208,494 and the number of missing vaccinations was 47,494.5. The capacities required to make up the missing vaccinations seem to be small, given that in Hungary, 4,746,822 adults have been vaccinated at the time of our study.

It is probable that the vaccination inequality demonstrated by our study will increase the intensity of the fourth wave of the pandemic and result in health losses in Hungary, as it was observed in the second wave as area deprivation related COVID-19 mortality inequality. [35]

Strengths and Limitations

This investigation covered the entire country, which prevented selection bias. Because vaccination registration is compulsory in Hungary, misclassification of the vaccination status was also avoided.

The unique feature of the Hungarian health statistical system is that the segregated colonies populated mainly by Roma are defined in the census data, and health indicators can be computed for these geographical units. Since, the registration of ethnicity in Hungarian health care administration is prohibited as it is in other European countries as well; the main strength of our study design was that it could avoid the violation of personal rights by utilizing SC related opportunity.

The living place of adults could be misclassified if the registered residential address was in a SC but the real residential address was in a CA (or vice versa). The extent of this

residential place misclassification is not known. Therefore, the potential dilution of the observed RsVC could not be quantified. Consequently, the real difference between SCs and CAs is underestimated by the observed RsVC=0.643 (i.e. the real RsVC is less than 0.643). Furthermore, it must be acknowledged that this misclassification could be strong in certain SCs, where the segregation effect could be seriously underestimated. Therefore, there could be nonidentified high-risk SCs. Altogether, the segregation effect suggested by an RsVC of 0.643 is valid, and the identified high-risk SCs are real high-risk populations.

In this study, calculations for cVC, sVC and RsVC were made with the assumption that SC localization has not changed from the last Hungarian census collection. Since the most recent, census took place in 2011, both geographic boundaries and demographic composition of SCs and CAs may have changed due to internal migration. This effect can be evaluated having the data from the next census, which will be implemented in 2022.

Taking into consideration that SCs are dominated but not exclusively inhabited by Roma, and about three quarters of the Roma population does not reside in SCs, our findings of SC vaccination inequality cannot be extrapolated to characterize Roma vaccination inequality in general.

This study was not able to collect data on many factors that can affect vaccination coverage apart from age, sex, and exemption certificate eligibility [36]. A detailed exploration on the background of SC-specific vaccination's variability needs further study. The social, cultural, economic and/or environmental differences between settlements with vaccination inequalities in SCs and those without inequality could be confounding variables in the rates of vaccination in these communities. Thus, there must be further understandings of the ethnic makeup and social determinants of health in Hungarian SCs.

Implications

The detected inequality demonstrates the necessity of special intervention programs targeting SCs in Hungary. Clearly, the general program was not as effective in SCs. The general program considered elderly individuals, patients with chronic disease, and health care personnel as high-risk groups. It appears that the population living in SCs, comprised dominantly of Roma, is also a high-risk group. Therefore, SCs should also be considered a specialized target group, where the general vaccination program should be adapted.

Extending the high-risk group definition could help to mitigate the impacts of the epidemic. This reconsideration is supported by published positive experiences and elaborated methodologies in countries where vaccination organizations and monitoring are adapted to racial/ethnic minorities. [6,37-42]

It is also urgent to expand these methods in Hungary to reduce COVID-19 vaccination inequalities. If it is successful, then the approach could be used as a template for other inequality reductions. Otherwise, if a special intervention program is not implemented and applied, the lesson by the end of the pandemic will be that the COVID-19 epidemic (and other problems) can be managed without considering ethnic inequalities and accepting the associated additional health loss.

One of the seven objectives of the European Union is to “Increase effective equal access to adequate desegregated housing and essential services” showing that segregation is among the most important characteristics of European Roma. It is highly probable that segregated colonies of Roma in other European countries face similar problems demonstrated in Hungary. [29] Moreover, our observations could help to characterize inequities occurring in similarly segregated groups around the world. For example, in the United States, Native American populations on reservations closely resemble the geographic and ethnic isolation patters exhibited by the Roma in Hungary. Thus, understanding vaccination discrimination in

the Roma-populated SCs parallels the inaccess to healthcare and vaccination disparities of minority populations in the United States. [43,44]

Conclusions

The COVID-19 vaccination program was less effective in Hungarian SCs, populated mainly by Roma, than in the nonsegregated part of the settlements with SCs. Accordingly, the population of SCs can be considered a distinct high-risk group with respect to vaccination coverage. This inequality was weaker but significant among elderly individuals. On the other hand, there was not detectable disparities in all settlements, demonstrating that there were effective COVID-19 vaccination protocols in Hungary, which avoided SC related geographical inequality which can be an indicator of Roma vs. non-Roma discrimination.

Our findings suggest that within the SC population, elderly individuals, patients with chronic disease, and healthcare personnel should be considered as distinct target groups with adapted vaccination programs. The required adapted methodology could be based on the benchmarking of good practice by which vaccination rate disparities were avoided in many Hungarian settlements and on the experiences reported in other countries. The need for SC-adapted methodology should be as clear as the need for a special vaccination program for healthcare personal.

Acknowledgements

This research would not have been possible without the IT support provided by Tibor Jenei (Department of Public Health and Epidemiology, Faculty of Medicine, University of Debrecen, Debrecen, Hungary), Zsófia Falusi (National Health Insurance Fund, Budapest, Hungary), and László Pál (National Health Insurance Fund, Budapest, Hungary).

References

1 Crawford R, Rutz DC, Evans DP. ‘Between Combat boots and Birkenstocks’–Lessons from HIV/AIDS, SARS, H1N1 and Ebola. *Public Health*. 2016; **141**:186–191. doi: 10.1016/j.puhe.2016.09.018.

2 Tam T. Fifteen years post-SARS: Key milestones in Canada’s public health emergency response. *Canada Commun Dis Rep*. 2018; **44**:98–101. doi: 10.14745/ccdr.v44i05a01.

3 Braden CR, Dowell SF, Jernigan DB, Hughes JM. Progress in global surveillance and response capacity 10 years after severe acute respiratory syndrome. *Emerg Infect Dis*. 2013; **19**:864–9. doi: 10.3201/eid1906.130192.

4 WHO. Coronavirus disease (COVID-19) Weekly Epidemiological Update and Weekly Operational Update. World Health Organization (WHO). 2021; https://www.who.int/emergencies/diseases/novel-coronavirus-2019/situation-reports. Accessed at 30/09/2021

5 Lo CH, Nguyen LH, Drew DA, Warner ET, Joshi AD, Graham MS, et al. Race, ethnicity, community-level socioeconomic factors, and risk of COVID-19 in the United States and the United Kingdom. *EClinicalMedicine*. 2021; **38**:101029. doi: 10.1016/j.eclinm.2021.101029.

6 Ayers CK, Kondo KK, Williams BE, Kansagara D, Advani SM, Smith M, et al. Disparities in H1N1 Vaccination Rates: a Systematic Review and Evidence Synthesis to Inform COVID-19 Vaccination Efforts. *J Gen Intern Med*. 2021; **36**:1734–1745. doi: 10.1007/s11606-021-06715-7.

7 Sze S, Pan D, Nevill CR, Gray LJ, Martin CA, Nazareth J, et al. Ethnicity and clinical outcomes in COVID-19: A systematic review and meta-analysis. *EClinicalMedicine*. 2020; **29**:100630. doi: 10.1016/j.eclinm.2020.100630.

8 Glance LG, Thirukumaran CP, Dick AW. The unequal burden of COVID-19 deaths in counties with high proportions of black and hispanic residents. *Med Care*. 2021; **59**:470–476. doi: 10.1097/MLR.0000000000001522.

9 Price-Haywood EG, Burton J, Fort D, Seoane L. Hospitalization and Mortality among Black Patients and White Patients with Covid-19. *N Engl J Med*. 2020; **382**:2534–2543. doi: 10.1056/NEJMsa2011686.

10 Parpia A, Pandey A, Martinez I, El-Sayed A, Wells C, Myers L, et al. Racial disparities in COVID-19 mortality across Michigan, United States. *EClinicalMedicine*. 2020; **33**:100761. doi: 10.1016/j.eclinm.2021.100761.

11 Seligman B, Ferranna M, Bloom DE. Social determinants of mortality from COVID-19: A simulation study using NHANES. *PLoS Med*. 2021; **18**:e1003490. doi: 10.1371/journal.pmed.1003490.

12 O’Dowd A. Covid-19: Black people over 80 in England are half as likely as white people to have been vaccinated. *BMJ*. 2021; **372**:n357. doi: 10.1136/bmj.n357.

13 WHO Feature stories: The Pfizer BioNTech (BNT162b2) COVID-19 vaccine: What you need to know. https://www.who.int/news-room/feature-stories. Accessed at 30/09/2021

14 Razieh C, Zaccardi F, Islam N, Gillies CL, V. Chudasama Y, Rowlands A, et al. Ethnic minorities and COVID-19: examining whether excess risk is mediated through deprivation. *Eur J Public Health*. 2021; **31**:630-634. doi: 10.1093/eurpub/ckab041.

15 Nguyen LH, Joshi AD, Drew DA, Merino J, Ma W, Lo C-H, et al. Racial and ethnic differences in COVID-19 vaccine hesitancy and uptake. *medRxiv*. 2021; doi: 10.1101/2021.02.25.21252402.

16 MacKenna B, Curtis HJ, Morton CE, Evans SJ, Smeeth L, OpenSAFELY Collaborative Tea et al. Trends, regional variation, and clinical characteristics of COVID-19

vaccine recipients: a retrospective cohort study in 23.4 million patients using OpenSAFELY. 2021; doi: 10.1101/2021.01.25.21250356. Accesed at 30/09/2021

17 Rostila M, Cederström A, Wallace M, Brandén M, Malmberg B, Andersson G. Disparities in Coronavirus Disease 2019 Mortality by Country of Birth in Stockholm, Sweden: A Total-Population–Based Cohort Study. *Am J Epidemiol*. 2021; **190**:1510–1518. doi: 10.1093/aje/kwab057.

18 Zalla LC, Martin CL, Edwards JK, Gartner DR, Noppert GA. A Geography of Risk: Structural Racism and Coronavirus Disease 2019 Mortality in the United States. *Am J Epidemiol*. 2021; **190**:1439–1446. doi: 10.1093/aje/kwab059.

19 Hughes MM, Wang A, Grossman MK, Pun E, Whiteman A, Deng L, et al. County-Level COVID-19 Vaccination Coverage and Social Vulnerability — United States, December 14, 2020-March 1, 2021. *MMWR Surveill Summ*. 2021; **70**:431-436. doi: 10.15585/mmwr.mm7012e1.

20 Brown CC, Young SG, Pro GC. COVID-19 vaccination rates vary by community vulnerability: A county-level analysis. *Vaccine*. 2021; **39**:4245–4249. doi: 10.1016/j.vaccine.2021.06.038.

21 Barry V, Dasgupta S, Weller DL, Kriss JL, Cadwell BL, Rose C, et al. Patterns in COVID-19 Vaccination Coverage, by Social Vulnerability and Urbanicity — United States, December 14, 2020–May 1, 2021. *MMWR Morb Mortal Wkly Rep*. 2021; **70**:818–824. doi: 10.15585/mmwr.mm7022e1.

22 Muhsen K, Na'aminh W, Lapidot Y, Goren S, Amir Y, Perlman S, et al. A nationwide analysis of population group differences in the COVID-19 epidemic in Israel, February 2020–February 2021. *Lancet Reg Health Eur*. 2021; 100130. doi: 10.1016/j.lanepe.2021.100130.

23 Duchen R, Iskander C, Chung H, Paterson J, Kwong J, Bronskill S, et al. The Role of a Resilient Information Infrastructure in COVID-19 Vaccine Uptake in Ontario. *Healthc Q*. 2021; **24**:7–11. doi: 10.12927/hcq.2021.26553.

24 Krieger N, Testa C, Hanage WP, Chen JT. US racial and ethnic data for COVID-19 cases: still missing in action. *The Lancet*. 2020; **396**(10261):e81. doi: 10.1016/S0140-6736(20)32220-0.

25 Kadambari S, Vanderslott S. Lessons about COVID-19 vaccine hesitancy among minority ethnic people in the UK. *Lancet Infect Dis*. 2021; **21**:1204–1206. doi: 10.1016/S1473-3099(21)00404-7.

26 Wong CA, Dowler S, Moore AF, Sosne EF, Young H, Tenenbaum JD, et al. COVID-19 Vaccine Administration, by Race and Ethnicity — North Carolina, December 14, 2020–April 6, 2021. *MMWR Morb Mortal Wkly Rep*. 2021; **70**:991–996. doi: 10.15585/mmwr.mm7028a2.

27 Griffin S. Covid-19: Ethnicity vaccination gap narrows in England, but concerns remain. *BMJ*. 2021; **372**:n505. doi: 10.1136/bmj.n505.

28 Nambi Ndugga, Latoya Hill, Samantha Artiga, Noah Parker: Latest Data on COVID-19 Vaccinations by Race/Ethnicity. Published: Jul 21, 2021; https://www.kff.org/coronavirus-covid-19/issue-brief/latest-data-on-covid-19-vaccinations-race-ethnicity/. Accessed at 30/09/2021

29 A Union of Equality: EU Roma strategic framework for equality, inclusion and participation. COM(2020) 620 final. https://eur-lex.europa.eu/legal-content/EN/TXT/HTML/?uri=CELEX:52020DC0620&from=EN. Accesed at 30/09/2021

30 Ádány R. Roma health is global ill health. *Eur J Public Health*. 2013**; 24**:702–3. doi: 10.1093/eurpub/cku143.

31 Pásztor, I.Z.; Pénzes, J.; Pálóczi, Á. The number and spatial distribution of the Roma population in Hungary—In the light of different approaches. *Folia Geogr*. 2016; **58**:5–21.

32 Sándor J, Kósa Z, Boruzs K, Boros J, Tokaji I, McKee M, et al. The decade of Roma Inclusion: did it make a difference to health and use of health care services? *Int J Public Health*. 2017; **62**:803–15. **62**(7):803-815. doi: 10.1007/s00038-017-0954-9.

33 Sándor J, Pálinkás A, Vincze F, Kovács N, Sipos V, Kőrösi L, et al. Healthcare utilization and all-cause premature mortality in Hungarian segregated Roma settlements: Evaluation of specific indicators in a cross-sectional study. *Int J Environ Res Public Health*. 2018; **15**:1835. doi: 10.3390/ijerph15091835.

34 Kósa K, Daragó L, Ádány R. Environmental survey of segregated habitats of Roma in Hungary: A way to be empowering and reliable in minority research. *Eur J Public Health*. 2011; **21**:463–8. doi: 10.1093/eurpub/ckp097.

35 Oroszi B, Juhász A, Nagy C, Horváth JK, McKee M, Ádány R. Unequal burden of COVID-19 in Hungary: a geographical and socioeconomic analysis of the second wave of the pandemic. *BMJ Glob Health*. 2021; **6**:e006427. doi: 10.1136/bmjgh-2021-006427.

36 Lee SQ, Raamkumar AS, Li J, Cao Y, Witedwittayanusat K, Chen L, Theng YL. Reasons for Primary Medication Nonadherence: A Systematic Review and Metric Analysis. *J Manag Care Spec Pharm*. 2018; 24(8):778-794. **24**:778–794. doi: 10.18553/jmcp.2018.24.8.778.

37 Chu H, Liu S. Light at the end of the tunnel: Influence of vaccine availability and vaccination intention on people's consideration of the COVID-19 vaccine. *Soc Sci Med*. 2021; **286**:114315. doi: 10.1016/j.socscimed.2021.114315.

38 Opel DJ, Lo B, Peek ME. Addressing Mistrust About COVID-19 Vaccines Among Patients of Color. *Annals of Internal Medicine*. 2021; **174**:698–700. doi: 10.7326/M21-0055.

39 Razai MS, Kankam HKN, Majeed A, Esmail A, Williams DR. Mitigating ethnic disparities in covid-19 and beyond. *BMJ*. 2021; **372**:m4921. doi: 10.1136/bmj.m4921.

40 Abdul-Mutakabbir JC, Casey S, Jews V, King A, Simmons K, Hogue MD, et al. A three-tiered approach to address barriers to COVID-19 vaccine delivery in the Black community. *The Lancet Global Health*. 2021; **9**:e749-e750. doi: 10.1016/S2214-109X(21)00099-1.

41 Ala A, Edge C, Zumla A, Shafi S. Specific COVID-19 messaging targeting ethnic minority communities. *EClinicalMedicine*. 2021; **35**:100862. doi: 10.1016/j.eclinm.2021.100862.

42 Darko J. Addressing the elephant in the room: COVID-19 vaccine hesitancy in Black and Asian communities. *Br J Gen Pract*. 2021; **71**:170. doi: 10.3399/bjgp21X715433.
. Accessed at 30/09/2021

43 Evans, MK. Covid's Color Line — Infectious Disease, Inequity, and Racial Justice. *N Engl J Med*., 2020; **383**:408-410. doi: 10.1056/NEJMp2019445.

44 Curtice K, Esther C. Indigenous Populations: Left Behind in the COVID-19 Response. *The Lancet (British Edition).* 2020; **395**:1753. doi: 10.1016/S0140-6736(20)31242-3.

Funding

This study was carried out in the framework of the " Routine monitoring for the health status and health care use in the Hungarian segregated colonies" program (BM/6327-3/2021, FEIF/951/2021-ITM), supported by the Deputy State Secretariat for Social Inclusion, Ministry of Interior.

Figure1

Click here to access/download;Figure;Figure1.tiff

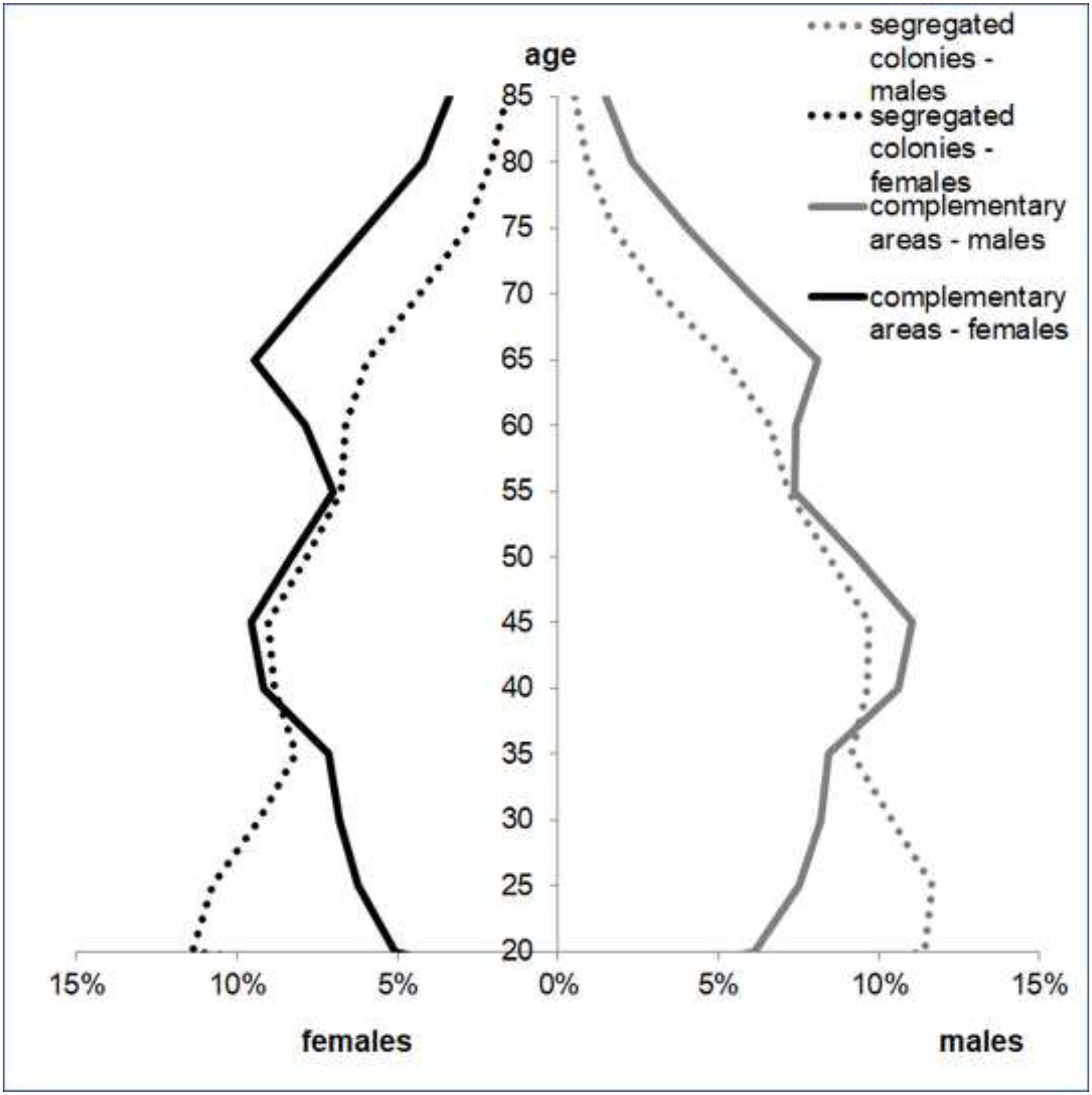

Figure2 Click here to access/download;Figure;Figure2.tiff

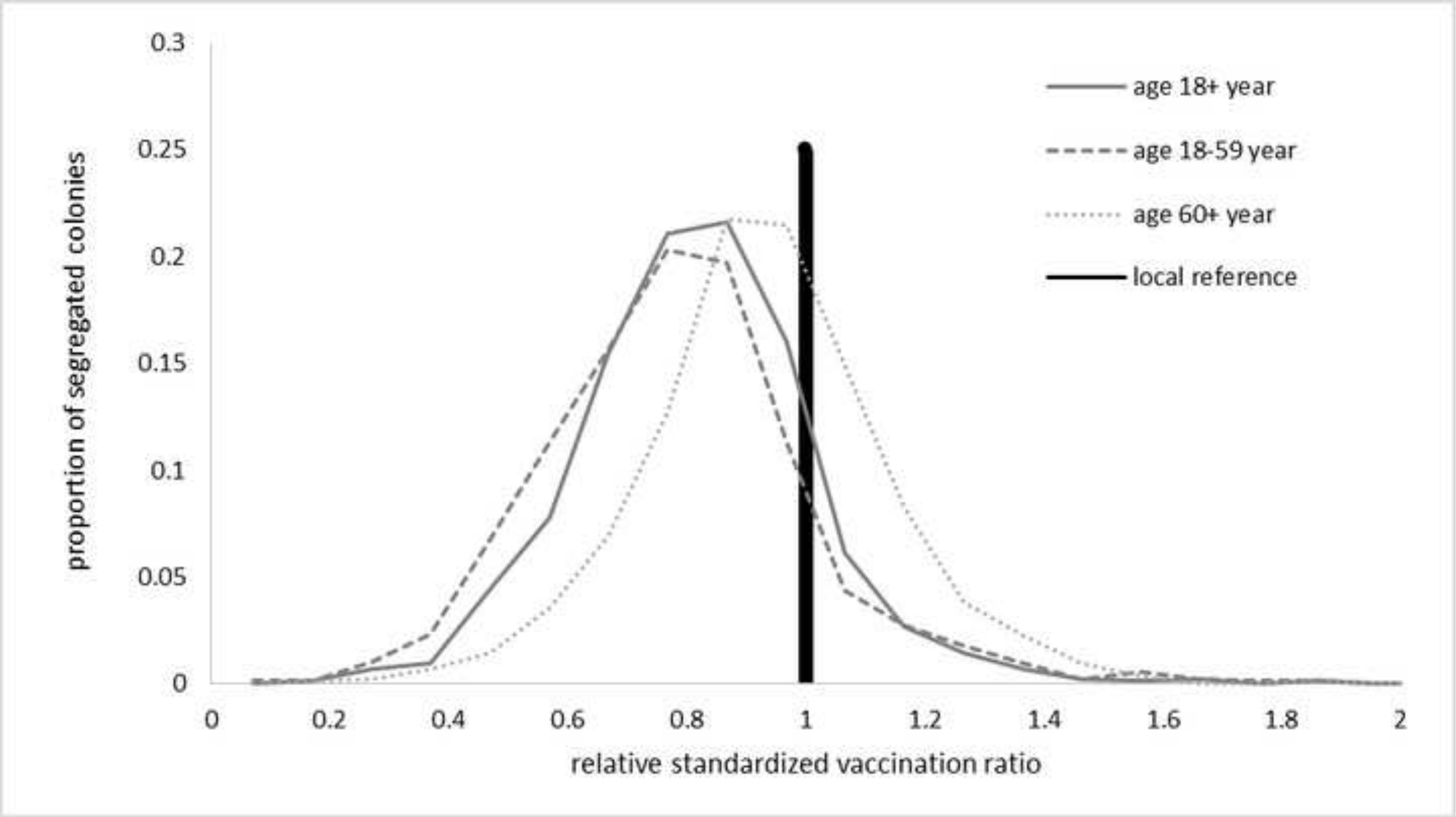

Click here to access/download;Figure;Figure3a.tif

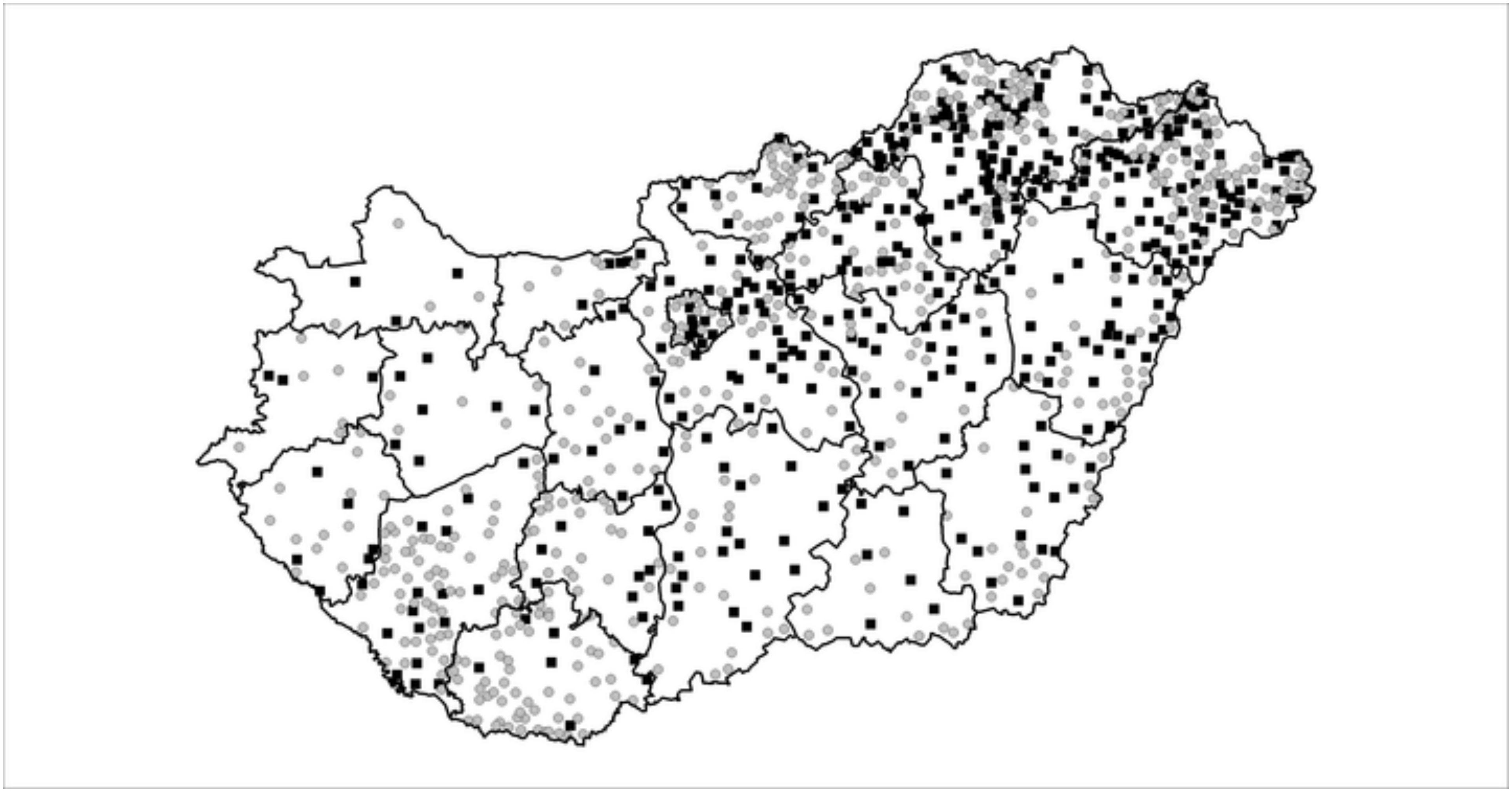

Figure3b

Click here to access/download;Figure;Figure3b.tif

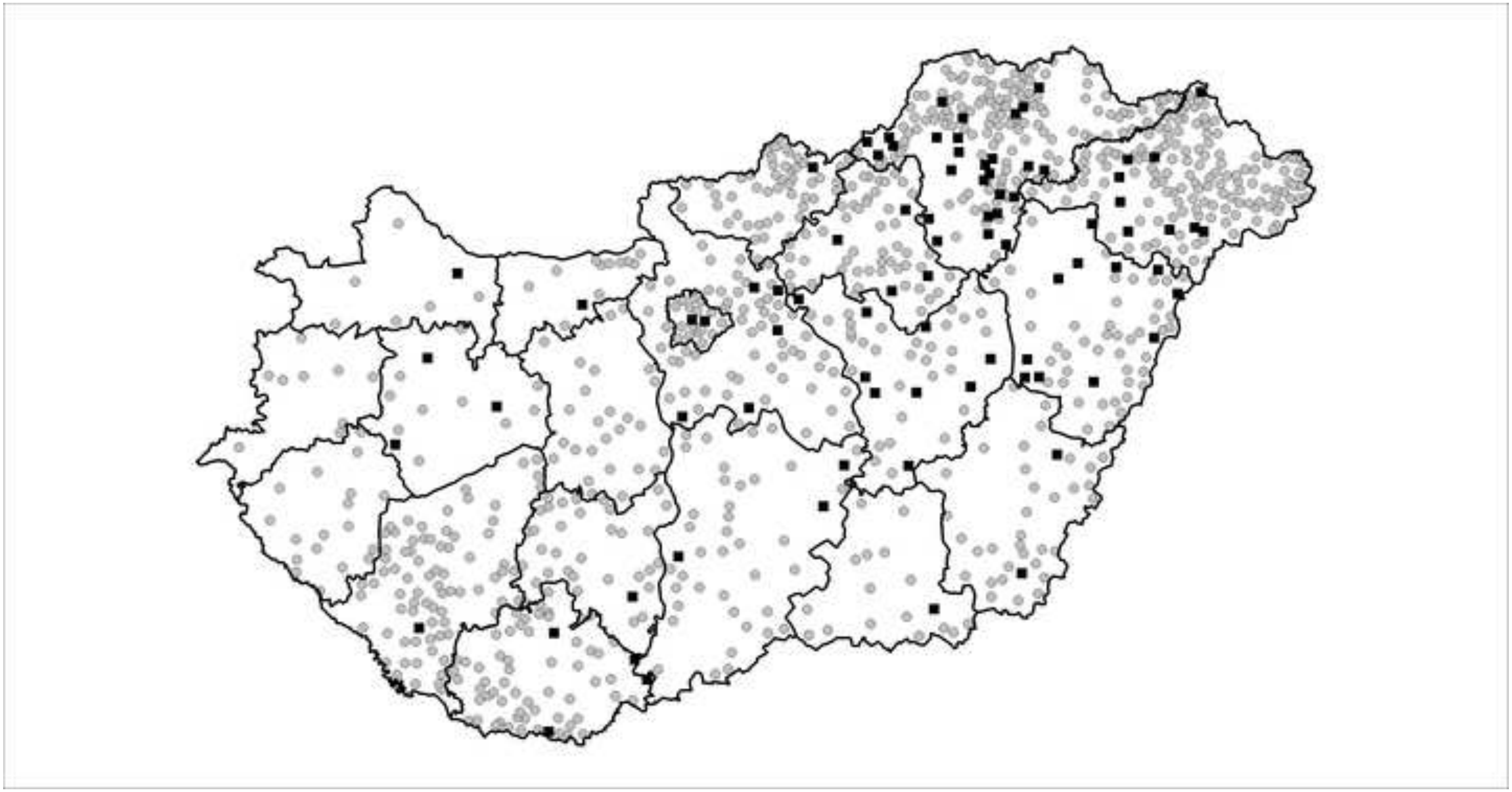

Figure3c

Click here to access/download;Figure;Figure3c.tif

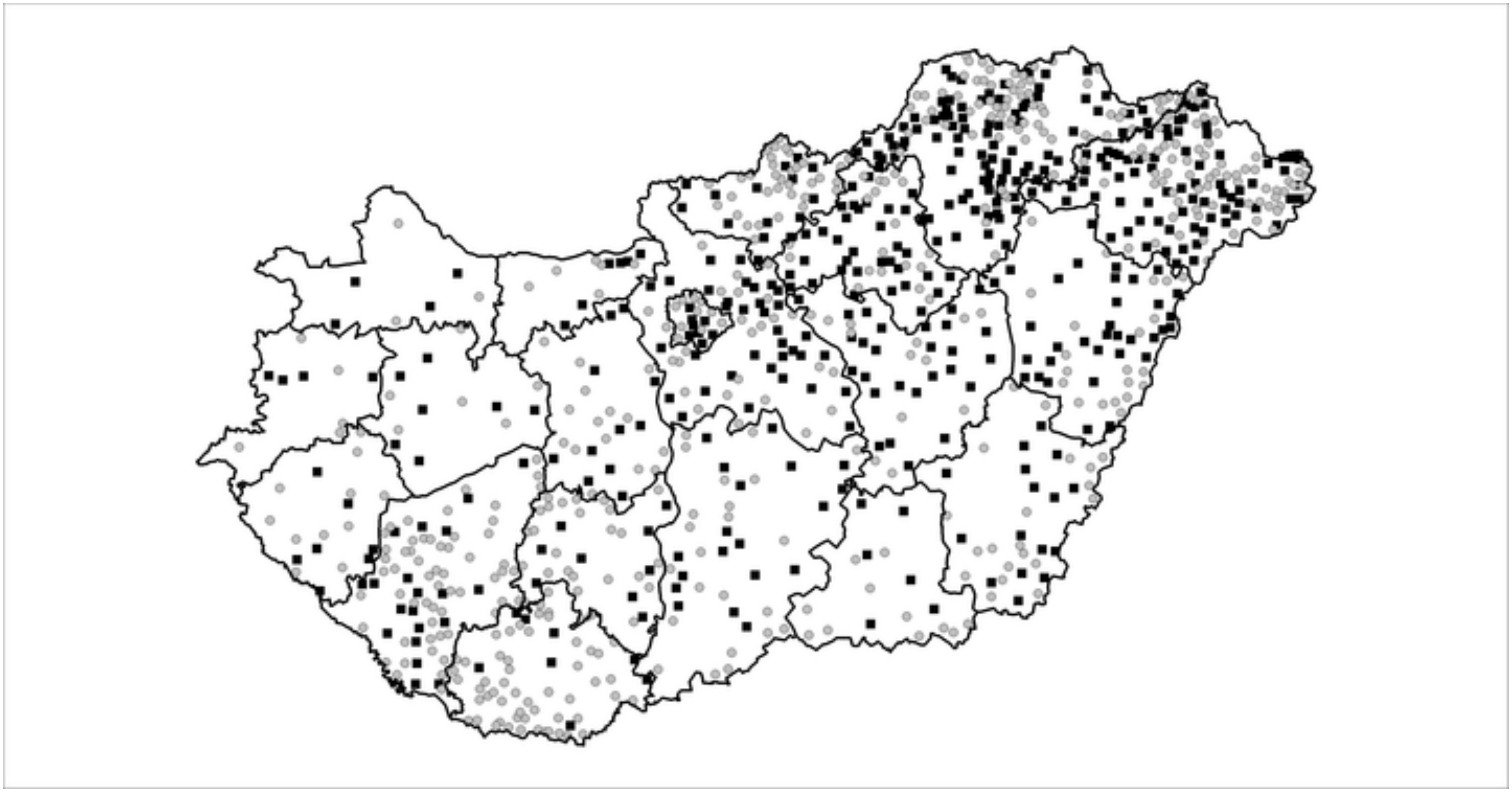

Supplement: Attachment — Submitted filename: PONE-D-21-32644_reviewer.pdf [file pone.0264363.s002.pdf]
