# Supplementary material for: COVID-19 vaccination coverage in deprived populations living in segregated colonies: A nationwide cross-sectional study in Hungary
Source: PLoS One. 2022 Feb 28;17(2):e0264363. doi: 10.1371/journal.pone.0264363 (PMC8884504; doi:10.1371/journal.pone.0264363)
Supplement: Attachment — Submitted filename: Response to Reviewer 2.docx [file pone.0264363.s004.docx]

Dear Editor and Reviewer,

Thank you very much for the careful second review of our manuscript. Please find enclosed the revised version of the manuscript “COVID-19 vaccination coverage in deprived populations living in segregated colonies: a nationwide cross-sectional study in Hungary” by János Sándor, et al.

Both comments have been considered. The corresponding changes made in the revised paper are summarized in our response after considering each of your suggestion. Answers along with the modifications we made are summarized below (comments/questions of Yours are in capitals).

Sincerely yours, Janos Sandor (on behalf of the authors)

Answers/reflections to the comments of the Editor:

1.

THERE IS A DISCREPANCY BETWEEN THE FIRST LINES OF THE ABSTRACT AND THE REST OF THE TEXT OF THE RESEARCH. ALTHOUGH IT IS FURTHER ELABORATED WITHIN THE TEXT LATER, THE ABSTRACT DOES NOT SUPPORT THE ABOVE STATEMENTS. PLEASE REPHRASE YOUR ABSTRACT ACCORDINGLY, AS THE TEXT CURRENTLY SUGGESTS THAT THIS RESEARCH FOCUSES ON THE ROMA ETHNIC MINORITY (LIVING IN SCS AND CAS).

Thank you for this comment. The misleading sentences have been modified accordingly.

Original text:

“The largest ethnic minority in Hungary is Roma, comprising 8.7% of the population. A quarter of them live in segregated colonies (SCs). Their health care use is restricted in many respects.”

Corrected version:

“The segregated colonies (SCs) in Hungary are populated mainly but not exclusively by Roma. Their health care use is restricted in many respects.”

2.

FURTHERMORE, WITH REGARDS TO LINE 298 NO REFERENCE OR SCIENTIFIC EVIDENCE HAS BEEN ADDED TO SUPPORT THE STATEMENT THAT ROMA AND BLACK/ASIAN COMMUNITIES FACE THE SAME CHALLENGES AND OBSTACLES IN TERMS OF THEIR ACCESS TO HEALTH SERVICES. IN MY OPINION THIS PART SHOULD BE EITHER EXTENDED, OR REPHRASED.

As it is acknowledged in the paper our investigation could not investigate the background of SC-related inequality in COVID-19 vaccination in Hungary. The demonstration of this inequality was aimed.

We referred the successful experiences with monitoring based interventions in the field of racial inequalities’ management in order to demonstrate that the basic discipline of public health is working in practice.

We could not undertake a deeper analysis of similarities and differences between racial minorities in the USA/UK and Hungarian segregated colonies populated mainly by Roma.

To clarify this intention, the paragraph has been rephrased.

Original text:

“Extending the high-risk group definition could help to mitigate the impacts of the epidemic on disadvantaged ethnic minorities. This reconsideration is supported by published positive experiences and elaborated methodologies in countries where vaccination organizations and monitoring are adapted to racial/ethnic minorities [6,43-48].”

Corrected version:

“Extending the high-risk group definition in Hungary could help to mitigate the impacts of the epidemic on disadvantaged, mainly Roma minority of SCs. Although, there are obvious differences between Hungarian SC inhabitants and the ethnic minorities of the United States and the United Kingdom, this reconsideration is supported by published positive experiences and elaborated methodologies in these countries where vaccination organizations and monitoring are adapted to racial/ethnic minorities [6,43-48].”
